# Supplementary material for: Influenza infection rewires energy metabolism and induces browning features in adipose cells and tissues
Source: Commun Biol. 2020 May 14;3:237. doi: 10.1038/s42003-020-0965-6 (PMC7224208; doi:10.1038/s42003-020-0965-6)
Supplement: Supplementary file 1 — Supplementary Information [file 42003_2020_965_MOESM1_ESM.pdf]

Supplementary Figures

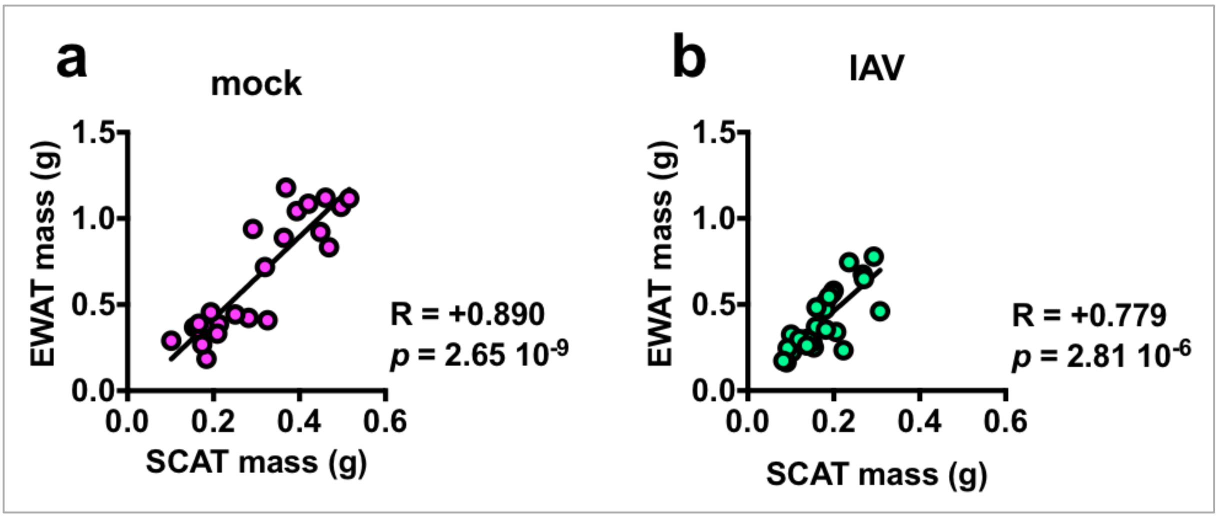

Supplementary Fig. 1. Correlation between the mass of adipose tissue depots (subcutaneous (SCAT) and epididymal (EWAT)), in mock-treated and IAV-infected mice. Spearman's correlation coefficients (and associated *p* values) of SCAT mass with EWAT mass in, **a** mock-treated, and **b** IAV-infected mice, 7 dpi. Each point represents one mouse, n = 25 mock-treated animals and n = 26 IAV-infected animals.

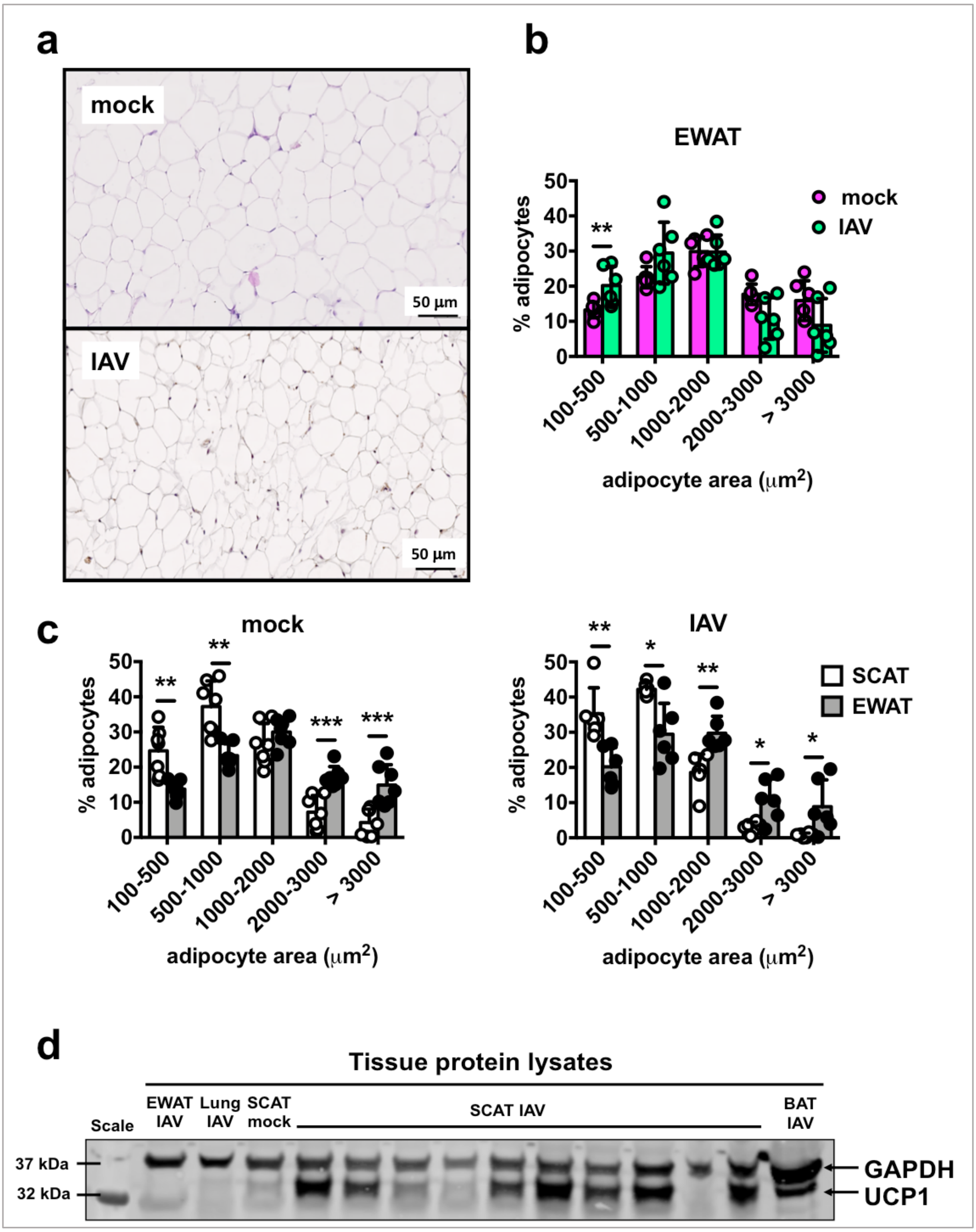

Supplementary Fig. 2. Comparison of adipocyte sizes in adipose tissue depots (subcutaneous (SCAT) and epididymal (EWAT)) from mock-treated and IAV-infected mice. **a** Representative images of hematoxylin/eosin staining of EWAT sections from mock-treated and IAV-infected mice at 7 dpi, n = 7 mock-treated animals and n = 7 IAV-infected animals.

b Frequency distributions (%) of adipocyte sizes (areas, μm^2) in the EWAT from mock-treated and IAV-infected mice, 7 dpi, n = 6 mock-treated animals and n = 6 IAV-infected animals. Individuals values, as well as means ± SD are shown. $^{**}p<0.01$. **c** Comparison of the frequency distributions (%) of adipocyte sizes (areas, μm^2) between the SCAT and the EWAT from mock-treated (mock) or from IAV-infected (IAV) mice, 7 dpi, n = 6 mock-treated animals and n = 6 IAV-infected animals. Individuals values, as well as means ± SD are shown. $^{*}p<0.05$, $^{**}p<0.01$, $^{***}p<0.001$. **d** Uncropped, full image of the western-blot of UCP1 shown on Fig. 2c. Lanes were loaded with EWAT, lung, SCAT and brown adipose tissue (BAT) protein lysates from mock-treated and IAV-infected mice at 7 dpi. GAPDH was used as an internal loading control.

Differences between mock-treated and IAV-infected groups (**b**), or between the SCAT and the EWAT in the mock-treated or in the IAV-infected conditions (**c**) were considered significant when $p<0.05$.

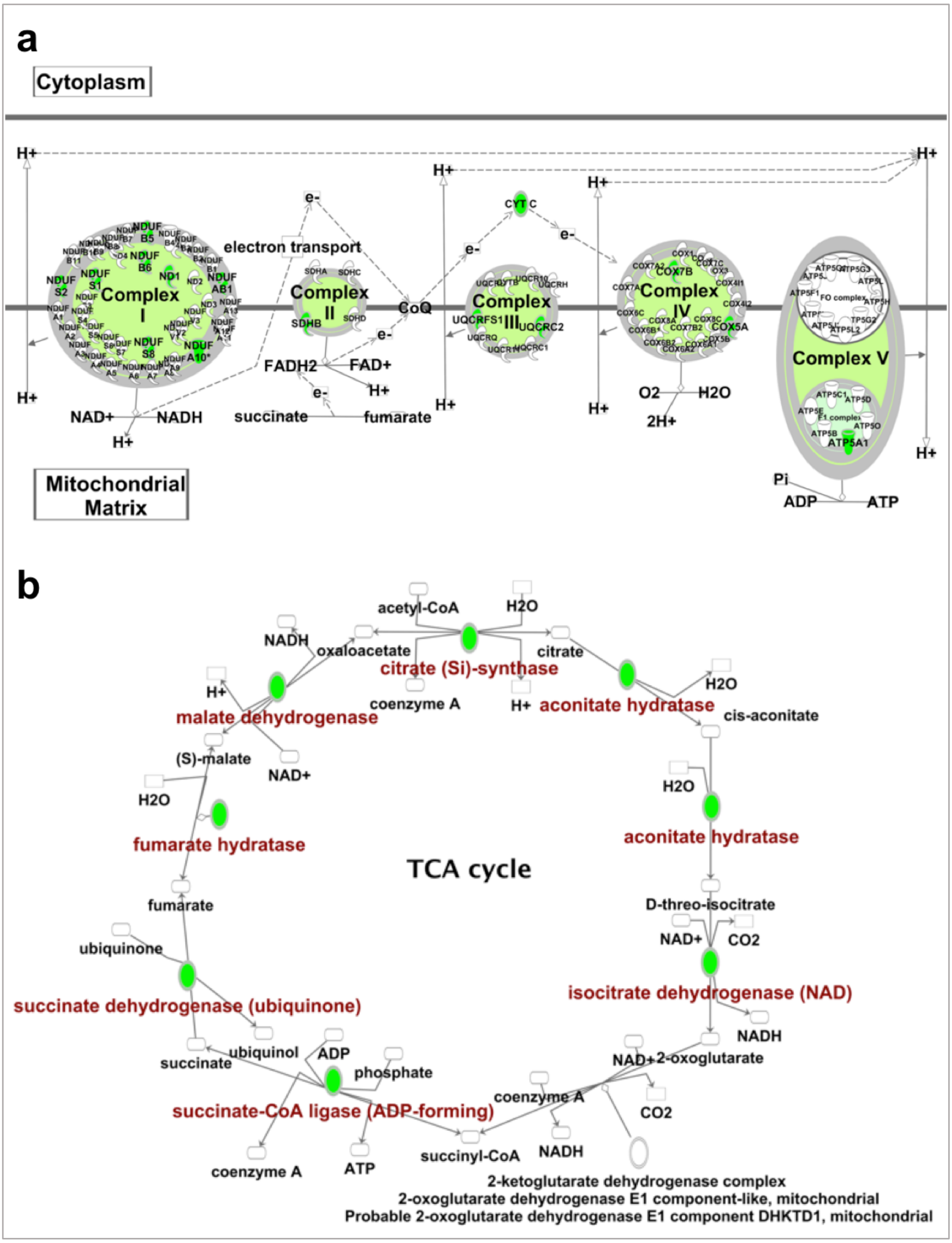

Supplementary Fig. 3. Influenza infection is associated with decreased mitochondrial fuel utilization and respiration in the subcutaneous adipose depots (SCAT). **a** Electron transport chain, and **b** Tricarboxylic acid (TCA) cycle, were constructed in the Ingenuity Pathway

Analysis (IPA) program. The pathways are colored based on a cut-off of an absolute correlation coefficient higher than 0.3. Green indicates a negative correlation.

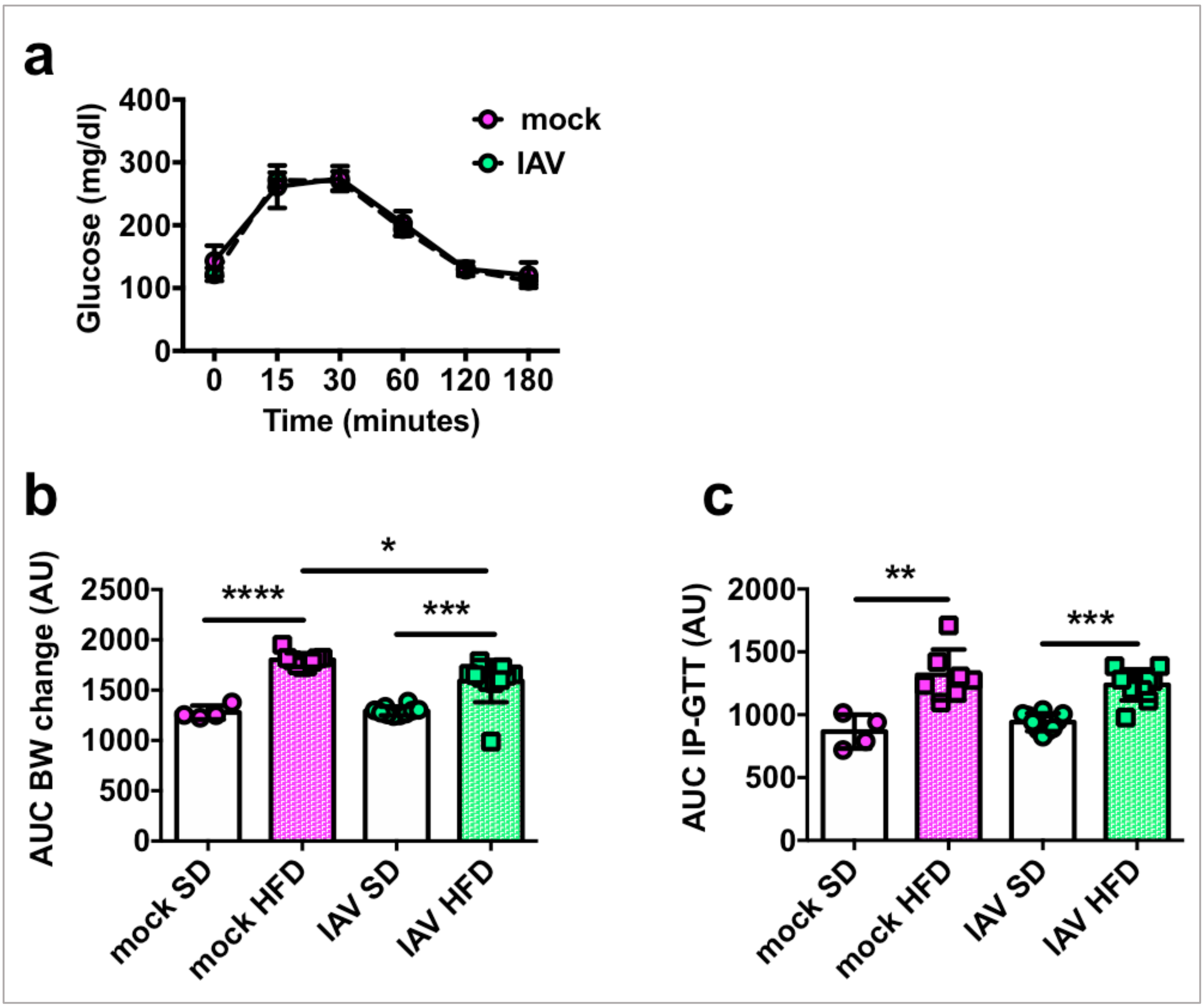

Supplementary Fig. 4. a Impact of influenza infection on glucose tolerance at distance from infection. Intraperitoneal glucose tolerance test (IP-GTT) was performed in 10-12 hours-fasted mock-treated and IAV-infected mice at 12 weeks post-infection. Blood samples for glucose levels determination were taken from the tail vein before glucose administration (i.p. injection, 1 g/kg BW), and after 15, 30, 60, 120 and 180 minutes, n = 8 mock-treated animals and n = 17 IAV-infected animals. Data are expressed as mean ± SD. **b** Area under the curve (AUC, expressed in arbitrary units (AU)) of % body weight change of mock-treated and IAV-infected mice fed with standard diet (SD) or with high-fat diet (HFD) from 7 dpi (see Fig. 4c), n = 5 mock-treated SD-fed animals (mock SD), n= 10 IAV-infected SD-fed animals (IAV SD), n = 10 mock-treated HFD-fed animals (mock HFD) and n = 15 IAV-infected HFD-fed animals (IAV HFD). Individuals values, as well as means ± SD are shown. $^{*}p<0.05$,

$^{***}p<0.001$, $^{****}p<0.0001$. **c** Area under the curve (AUC, expressed in arbitrary units (AU)) of the intraperitoneal glucose tolerance test performed in mock-treated and IAV-infected mice fed with standard diet (SD) or with high-fat diet (HFD), 18 weeks post-infection (see Fig. 4d), n = 5 mock-treated SD-fed animals (mock SD), n = 10 IAV-infected SD-fed animals (IAV SD), n = 9 mock-treated HFD-fed animals (mock HFD) and n = 12 IAV-infected HFD-fed animals (IAV HFD). Individuals values, as well as means ± SD are shown. $^{**}p<0.01$, $^{***}p<0.001$.

Differences between mock-treated and IAV-infected groups (**a**, **b**, **c**), or between SD-fed and HFD-fed groups (**b**, **c**) were considered significant when $p<0.05$.

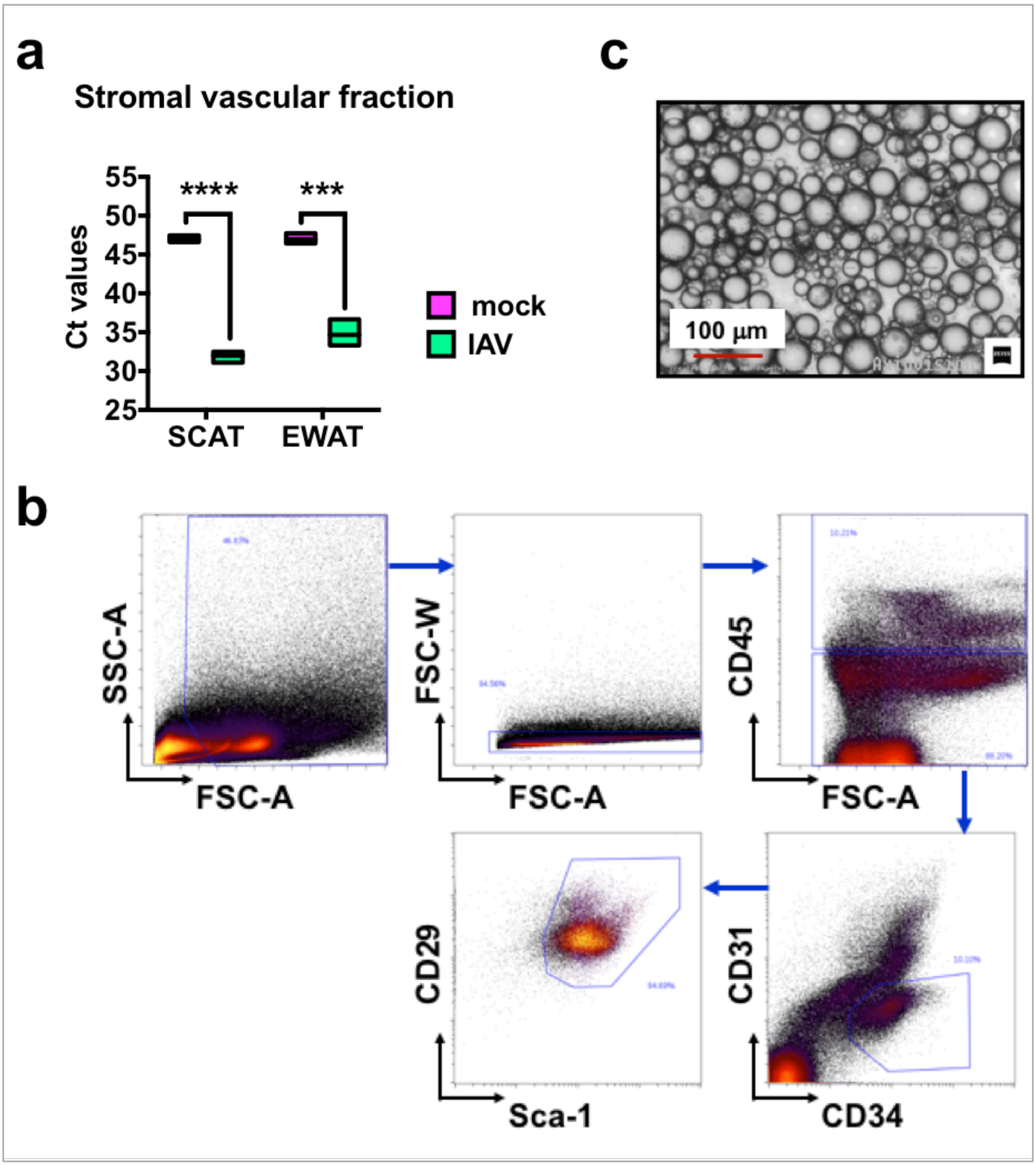

Supplementary Fig. 5. a Viral RNA levels (expressed as cycle threshold (Ct) values) in stromal vascular fraction (SVF) cells isolated from the subcutaneous (SCAT) and the epididymal (EWAT) adipose depots of mock-treated and IAV-infected mice at 7 dpi, n = 3 mock-treated animals and n= 3 IAV-infected animals. $^{***}p<0.001$, $^{****}p<0.0001$. **b** Gating strategy used for the sorting of preadipocytes and immune cells from the adipose tissues of mock-treated and IAV-infected mice. SVF cell suspensions were prepared from the SCAT and EWAT of mock-treated and IAV-infected mice (individually), 7 dpi, n = 3 mock-treated

animals and n = 6 IAV-infected animals. Immune cells were identified as CD45^{+} cells. Preadipocytes were identified as CD45^{-} CD31^{-} CD29^{+} CD34^{+} Sca1^{+} cells. **c** Representative picture of primary adipocyte preparations from the adipose tissues (Axiovert 40C, Zeiss). Differences between mock-treated and IAV-infected groups (**a**) were considered significant when $p<0.05$.

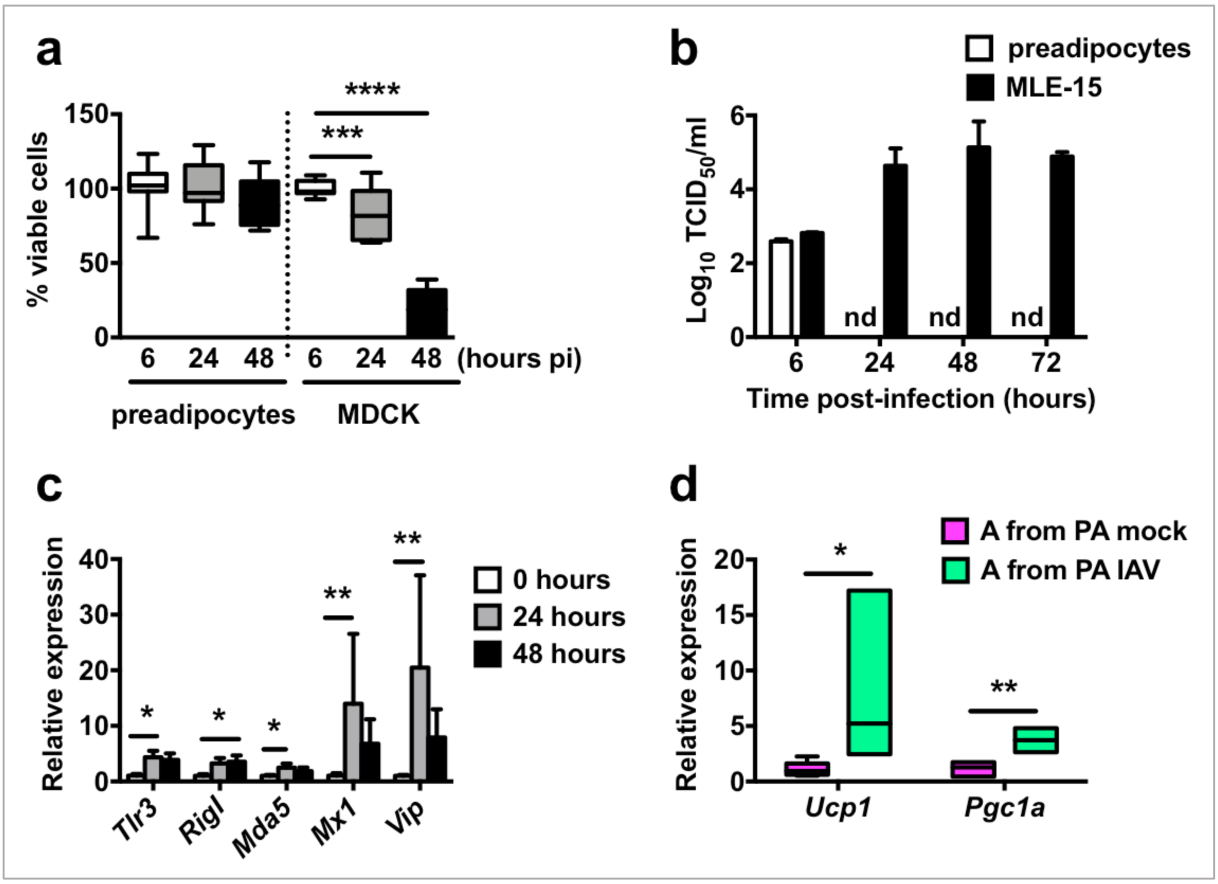

Supplementary Fig. 6. a Assessment of the viability of IAV-infected mouse preadipocytes. Preadipocytes (3T3-L1 cells) and Madin-Darby Canine Kidney (MDCK) cells were infected with IAV (MOI of 1) and cell viability was determined at 6, 24 and 48 hours post-infection (hpi), using the Orangu™ colorimetric assay. Shown are % of viable cells, n = 4 biologically independent samples per condition. $^{***}p<0.001$, $^{****}p<0.0001$. **b** Infectious particle release from infected murine preadipocytes and lung epithelial cells (MLE-15 cell-line). Cells were infected at MOI of 0.01, 0.25 and 0.5, and supernatants were collected at 6, 24, 48 and 72 hpi. Infectious viral titers ($TCID_{50}$/ml) of supernatants at MOI of 0.5 were determined by endpoint titration in MDCK cells, n = 2 biologically independent samples per condition (precluding any statistical analysis), nd = not detected. Data are expressed as mean ± SD. **c** Relative mRNA expression (RT-qPCR) of the antiviral innate-immune-related genes *Tlr3*, *RigI*, *Mda5*, *Mx1* and *Vip* in mock-treated and IAV-infected preadipocytes (3T3-L1 cell-line) (low MOI of 0.01, 24 and 48 hpi), n = 3 mock-treated biologically independent samples and n

= 4 IAV-infected biologically independent samples Data are expressed as mean ± SD. $^{*}p<0.05$, $^{**}p<0.01$. **d** Relative mRNA expression of *Ucp1* and *Pgc1a* in adipocytes differentiated from mock-treated preadipocytes or from IAV-infected preadipocytes (MOI of 1), n = 6 biologically independent samples per condition. $^{*}p<0.05$, $^{**}p<0.01$.

Differences between % viability at 6 hpi and at 24 or 48 hpi (**a**), and mock-treated and IAV-infected groups (**c**, **d**) were considered significant when $p<0.05$.

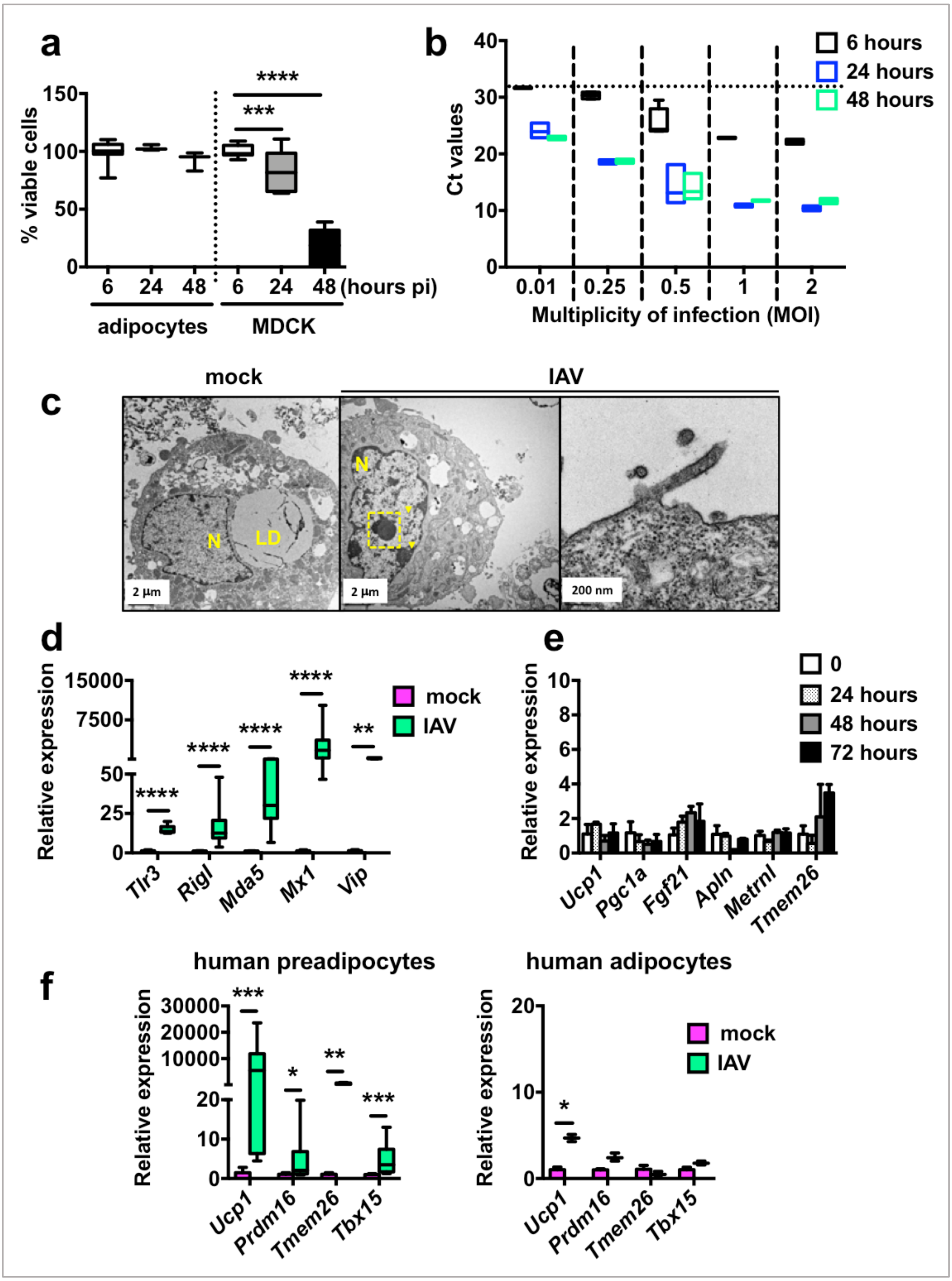

Supplementary Fig. 7. a Assessment of the viability of IAV-infected mouse adipocytes (differentiated from 3T3-L1 preadipocytes) and IAV-infected Madin-Darby Canine Kidney (MDCK) cells (MOI of 1), using the Orangu^{TM} colorimetric assay. Shown are % of viable

cells at 6, 24 and 48 hours post-infection (hpi), n = 4 biologically independent samples per condition. $^{***}p<0.001$, $^{****}p<0.0001$. **b** Viral RNA levels (expressed as cycle threshold (Ct) values) in IAV-infected adipocytes (MOI of 0.01, 0.25, 0.5, 1 or 2) at 6, 24 and 48 hpi, n = 4 biologically independent samples per condition. The dotted line indicates the 6 hpi/0.01 MOI mean Ct value. **c** Representative transmission electron microscopy sections of mock-treated and IAV-infected adipocytes (MOI of 5, 24 hpi), n = 3 biologically independent samples per condition. Classical nuclear (yellow arrowheads) and nucleolar (yellow dashed frame) fingerprints associated with IAV infection are shown. A detailed view of empty viral particles at budding regions is presented. N = nucleus, LD = lipid droplet. **d** Relative mRNA expression (RT-qPCR) of the viral-sensing genes *Tlr3*, *RigI*, *Mda5*, *Mx1* and *Vip* in mock-treated and IAV-infected adipocytes (MOI of 1, 24 hpi). For *Tlr3* and *Vip*: n = 5 biologically independent samples per condition. For *RigI*, *Mda5* and *Mx1*: n = 7 biologically independent mock-treated samples and n = 18 biologically independent IAV-infected samples. $^{**}p<0.01$, $^{****}p<0.0001$. **e** Relative mRNA expression of the browning-like/beiging-related genes *Ucp1*, *Pgc1a*, *Fgf21*, *Apln*, *Metrnl* and *Tmem26* in mock-treated and IAV-infected adipocytes at 24, 48 and 72 hpi (MOI of 1), n = 6 biologically independent samples per condition. Data are expressed as mean ± SD. **f** Relative mRNA expression of genes involved in the browning-like/beiging process (*Ucp1*, *Prdm16*, *Tmem26* and *Tbx15*) in mock-treated and IAV-infected primary human preadipocytes, and in mock-treated and IAV-infected human adipocytes (differentiated from primary human preadipocytes) (MOI of 1, 48 hpi), n = 10 biologically independent mock-treated preadipocyte samples, n = 6 biologically independent IAV-infected preadipocyte samples, n = 5 biologically independent mock-treated adipocyte samples and n = 6 biologically independent IAV-infected adipocyte samples. $^{*}p<0.05$, $^{**}p<0.01$, $^{***}p<0.001$. Differences between mock-treated and IAV-infected groups (**c**, **e**) were considered significant when $p<0.05$.

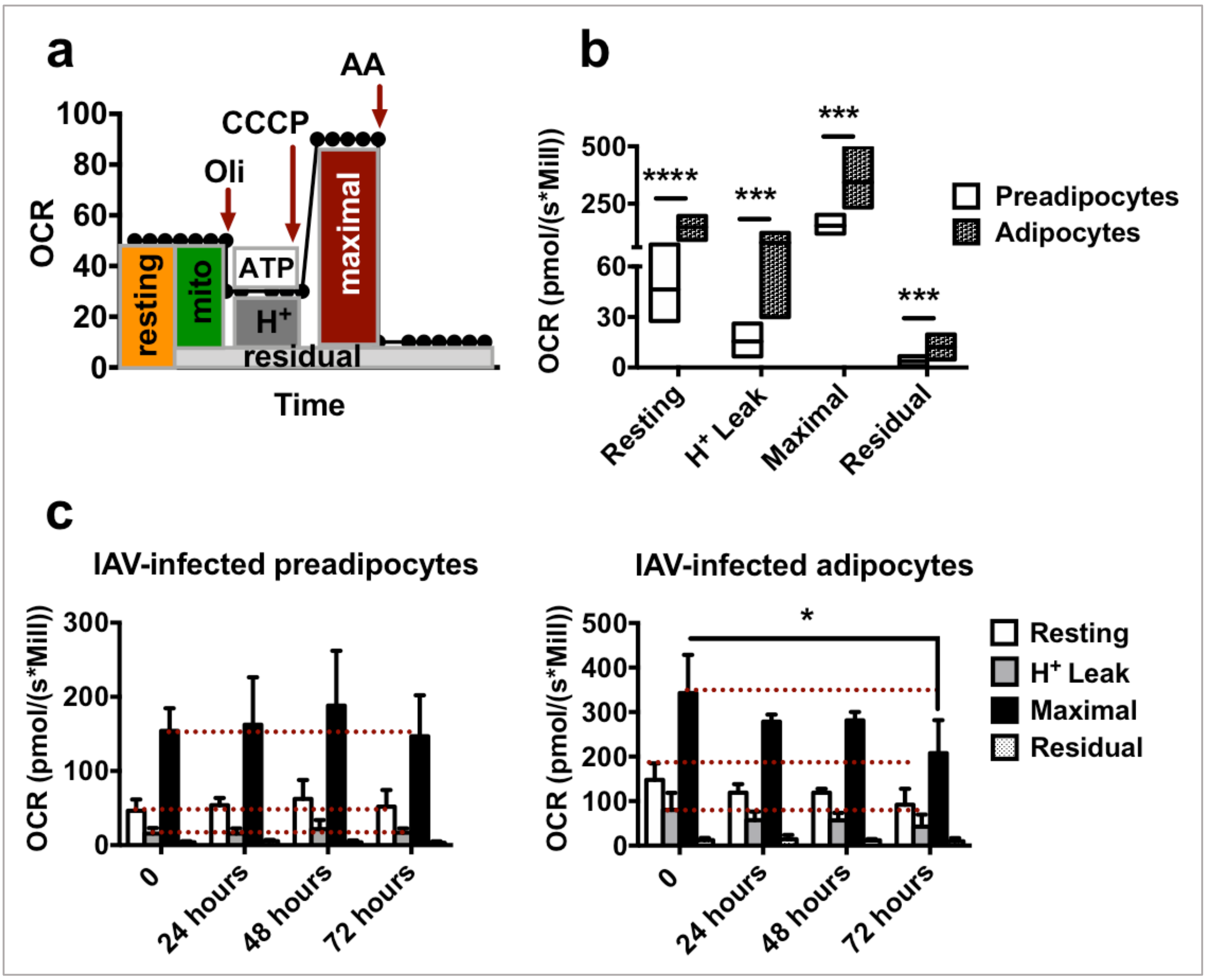

Supplementary Fig. 8. Influenza virus infection-induced changes in mitochondrial bioenergetics are different in preadipocytes and adipocytes. **a** Scheme showing dissection of the Oxygen consumption rates (OCRs) in response to, successively, the ATP synthase inhibitor: oligomycin (Oli), the mitochondrial uncoupler: carbonyl cyanide 3-chlorophenylhydrazone (CCCP), and antimycin A (AA). **b** After recording of resting respiration in mock-treated preadipocytes (3T3-L1 cells) and adipocytes (differentiated from 3T3-L1 preadipocytes), the addition of Oli allowed to identify the proportion of resting respiration that contributed to H^+ leak. Maximal and residual cellular respiratory capacities were obtained after the addition of, respectively, CCCP and AA, n = 8 independent experiments. OCRs are expressed in pmole O_2/second*million cells. $^{***}p<0.001$, $^{****}p<0.0001$. **c** Resting, H^+ leak, maximal and residual respiratory capacities of mock-treated

and IAV-infected preadipocytes (3T3-L1 cells), and adipocytes (differentiated from 3T3-L1 preadipocytes) at 24, 48, and 72 hpi (MOI of 1), determined after the sequential addition of Oli, CCCP and AA, n = 8 independent experiments. OCRs are expressed as mean ± SD, in pmole O_2/second*million cells. $^{*}p<0.05$.

Differences between mock-treated preadipocytes and adipocytes (**b**), and between resting state OCR value and H^+ leak, maximal and residuals OCRs values (**c**) were considered significant when $p<0.05$.

Supplementary Tables

Upstream Regulator	Predicted Activation	*p*-value of overlap	Target molecules in dataset
STAT1	Activated	$6{,}16\ 10^{-46}$	Batf2,C4a/c4b,Cd274,Cebpd,Cmpk2,Cxcl9, Cyp2d9,Gbp2,Gbp3,Gbp4,Gbp5,Gbp6,Hlae, Ifi44,Ifi47,Ifit1b,Ifit2,Ifit3,Ifitm3,Igtp,Iigp1, Il18bp,Irf1,Irf7,Irf9,Irgm1,Isg15,Ly6e,Oas1, Oas2,Oasl2,Parp9,Psmb8,Psmb9,Rnf213, Rsad2,Rtp4,Samhd1,Serpina3g,Serping1, Slfn13,Socs1,Sp110,Stat1,Stat2,Tap1,Tgtp1/ Tgtp2,Tnfsf10,Trim21,Ubd,Usp18,Wars, Xaf1,Zbp1
IRF7	Activated	$8{,}42\ 10^{-44}$	Adar,Cmpk2,Daxx,Gbp3,Gbp4,Gbp5,Ifi44, Ifi47,Ifit1b,Ifit2,Ifit3,Ifitm3,Igtp,Irf1,Irf7, Irf9,Irgm,Irgm1,Isg15,Ly6a,Oas1,Oas2, Oasl2,Parp14,Phf11,Psmb8,Psmb9,Rsad2, Rtp4,Socs1,Stat1,Stat2,Tap1,Tap2,Tnfsf10, Trim21,Trim30a/Trim30d,Uba7,Usp18,Xaf1 Zbp1
IFNG	Activated	$2{,}19\ 10^{-41}$	Batf2,Bst2,C4a/C4b,Casp12,Ccl6,Cd163, Cd274,Cebpd,Chac1,Cmpk2,Cxcl9,Daxx, Depp1,Dtx3l,Dusp9,Fam107a,Fkbp5,Gbp2, Gbp3,Gbp4,Gbp5,Gbp6,Gbp7,Gbp8,Glul, Gvin1,H6pd,Hip1,HlaA,Hlae,Id1,Ifi44,Ifi47, Ifit1b,Ifit2,Ifit3,Ifitm3,Igtp,Iigp1,Il18bp,Il6r, Irf1,Irf7,Irf9,Irgm,Irgm1,Isg15,Lox,Ly6a, Ly6e,Man2a1,Mitf,Mt1,Nedd9,Ntrk2,Oas1, Oas2,Parp14,Parp9,Psmb8,Psmb9,Rsad2,Rtp 4,Samhd1,Serpina3g,Serpin1,Socs1,Sp110, Stat1,Stat2,Tap1,Tap2,Tgtp1/Tgtp2,Thbs1, Timp4,Tnfsf10,Trim21,Txnip,Ubd,Usp18, Wars,Xaf1,Zbtb16
IFNB1	Activated	$5{,}05\ 10^{-40}$	Apol11b,Bst2,Cd274,Cmpk2,Daxx,Gbp2, Gbp3,Gbp4,Gbp5,Gbp6,Gbp7,Gbp8,Gvin1, Hlaa,Ifi47,Ifit1b,Ifit2,Ifit3,Igtp,Irf1,Irf7,Irf9, Irgm,Irgm1,Isg15,Oas1,Oas2,Oasl2,Parp14, Pdk4,Rnasel,Rsad2,Slfn13,Socs1,Stat1,Stat2 Tgtp1/Tgtp2,Thbs1,Thbs2,Tnfsf10,Trim21, Trim30a/Trim30d,Uba7,Usp18,Xaf1,Zbp1
IRF3	Activated	$7{,}56\ 10^{-31}$	Adar,Ahnak,Bst2,Cd274,Cmpk2,Cxcl9,Dax x,Gbp5,Ifi44,Ifi47,Ifit1b,Ifit2,Ifit3,Ifitm3,Igt p,Irf1,Irf7,Irgm,Irgm1,Isg15,Oas1,Oas2,Oasl 2,Parp14,Phf11,Pnp,Rsad2,Stat1,Stat2,Tap1, Tnfsf10,Trim30a/Trim30d,Usp18,Zbp1

Supplementary Table 1. Top five upstream regulators and their target molecules identified by Ingenuity pathway analysis (IPA) for the differentially expressed genes (DEGs) upregulated both in the subcutaneous adipose tissue (SCAT) and the epididymal adipose tissue (EWAT) of influenza-infected mice, relative to mock-treated animals (see Fig. 3b). IRF = interferon regulatory factor, IFNG = interferon gamma, IFNB = interferon beta.

Upregulated DEGs in SCAT only		
**Top Canonical Pathways**	***p* values**	**Overlap**
Axonal guidance signaling	1.33 10^{-05}	3.2% (16/501)
RhoA signaling	1.60 10^{-05}	6.5% (8/123)
VEGF signaling	7.89 10^{-05}	6.1% (7/114)
Regulation of actin-based motility by Rho	2.09 10^{-04}	6.4% (6/94)
Signaling by Rho family GTPases	5.91 10^{-04}	3.5% (9/259)
**Top Upstream Regulators**	***p* values**	**Predicted activation**
CEBPB	6.43 10^{-08}	Activated
TGFB1	1.27 10^{-07}	Activated
dihydrotestosterone	2.34 10^{-07}	Activated
beta-estradiol	8.98 10^{-07}	No prediction
GNA15	9.81 10^{-07}	No prediction
**Upregulated DEGs in EWAT only**		
**Top Canonical Pathways**	***p* values**	**Overlap**
Allograft rejection signaling	3.84 10^{-06}	7.0% (6/86)
OX40 signaling pathway	5.01 10^{-06}	6.7% (6/90)
Communication innate/adaptive immune cells	7.29 10^{-06}	6.2% (6/96)
Antigen presentation pathway	3.75 10^{-05}	10.3% (4/39)
Bladder cancer signaling	1.10 10^{-04}	5.2% (5/97)
**Top Upstream Regulators**	***p* values**	**Predicted activation**
Interferon alpha	4.96 10^{-19}	Activated
poly rI:rC-RNA	2.54 10^{-17}	Activated
STAT6	4.99 10^{-17}	No prediction
lipopolysaccharide	1.06 10^{-15}	Activated
IRF3	1.37 10^{-13}	Activated

Supplementary Table 2. Top five canonical pathways and upstream regulators identified by Ingenuity pathway analysis (IPA) for the differentially expressed genes (DEGs) upregulated either in the subcutaneous adipose tissue (SCAT) or in the epididymal adipose tissue (EWAT) after influenza infection, relative to mock-treated controls. The enriched pathways are ranked

by *p* values and percentages of the overlapping genes mapped against the total number of those in that pathway.

VEGF = vascular endothelial growth factor, CEBPB = CAAT/enhancer-binding protein beta, TGFB = transforming growth factor beta, GNA = Guanine nucleotide-binding protein subunit alpha, IRF = interferon regulatory factor.

Upstream Regulator	Predicted Activation	*p*-value of overlap	Target molecules in dataset
SCAP	Inhibited	1,71 10^{-33}	Acaca,Acly,Acss2,Cyp51a1,Dhcr7,Ehhadh, Elovl6,Fasn,Fdps,Idi1,Ighm,Insig1,Lss, Msmo1,Mvd,Nsdhl,Pmvk,Sc5d,Scd2,Sqle, Srebf1,Thrsp,Tm7sf2
SREBF2	Inhibited	3,41 10^{-30}	Acaca,Acly,Acss2,Cyp51a1,Dhcr7,Elovl6, Fasn,Fdps,Idh1,Idi1,Insig1,Lss,Msmo1,Mvd, Nsdhl,Pmvk,Sc5d,Sqle,Srebf1,Thrsp,Tm7sf2
SREBF1	Inhibited	7,20 10^{-26}	Acaca,Acly,Acss2,Cyp51a1,Dhcr7,Ehhadh, Elovl6,Fasn,Fdps,Idh1,Idi1,Ighm,Insig1,Lpl, Lss,Msmo1,Mvd,Nsdhl,Pmvk,Pnpla3,Sc5d, Scd2,Sqle,Srebf1,Thrsp,Tm7sf2
INSIG1	Activated	1,83 10^{-22}	Acaca,Acly,Acss2,Cyp51a1,Dhcr7,Elovl6, Fasn,Fdps,Idi1,Lpl,Lss,Mlxipl,Pmvk,Ptges, Scd2,Sqle,Srebf1,Tm7sf2
PPARA	No prediction	1,23 10^{-21}	Acaca,Acss2,Cdkn2c,Cth,Cyb5b,Cyp51a1, Dhcr7,Dio2,Ehhadh,Elovl6,Fasn,Fdps,Gpd1, Gpd2,Gpt,Idi1,Insig1,Lpl,Lss,Msmo1,Mvd, Nsdhl,Paqr9,Plin1,Pmvk,Sc5d,Sqle,Srebf1

Supplementary Table 3. Top five upstream regulators and their target molecules identified by Ingenuity pathway analysis (IPA) for the differentially expressed genes (DEGs) downregulated both in the subcutaneous adipose tissue (SCAT) and the epididymal adipose tissue (EWAT) after influenza infection, relative to mock-treated controls (see Fig. 3c). SCAP = SREBP cleavage-activation protein, SREBF (or SREBP) = sterol regulatory element-binding factor (or protein), INSIG = insulin-induced gene, PPARA = peroxisome proliferator-activated receptor alpha.

**Top Canonical Pathways**	***p* values**	**Overlap**
Interferon signaling	$2.46\ 10^{-17}$	36.1% (13/36)
IRF activation by cytosolic PRRs	$7.74\ 10^{-17}$	24.2% (15/62)
Role of PRRs in viruses and bacteria recognition	$8.54\ 10^{-14}$	12.4% (17/137)
Antigen presentation pathway	$1.25\ 10^{-13}$	28.9% (11/38)
Allograft rejection signaling	$4.28\ 10^{-12}$	15.5% (13/84)
**Top Upstream Regulators**	***p* values**	**Predicted activation**
IRF3	$4.77\ 10^{-95}$	Activated
Ifnar	$4.11\ 10^{-86}$	Activated
TRIM24	$3.30\ 10^{-76}$	Inhibited
IRF7	$3.99\ 10^{-76}$	Activated
STAT1	$2.82\ 10^{-62}$	Activated

Supplementary Table 4. Top five canonical pathways and upstream regulators identified by Ingenuity pathway analysis (IPA) for the differentially expressed genes (DEGs) upregulated both in preadipocytes and adipocytes after influenza infection, relative to mock-treated controls (see Fig. 7a). The enriched pathways are ranked by *p* values and percentages of the overlapping genes mapped against the total number of those in that pathway.

IRF = interferon regulatory factor, PRR = pattern recognition receptor, Ifnar = interferon-alpha/beta receptor alpha chain.

Top Canonical Pathways	*p* values	Overlap
Hereditary breast cancer signaling	2.24 10^{-04}	3.5% (5/142)
DNA double-strand break repair by homologous recombination	1.30 10^{-03}	14.3% (2/14)
Retinoic acid mediated apoptosis signaling	1.70 10^{-03}	4.9% (3/61)
GADD45 signaling	2.41 10^{-03}	10.5% (2/19)
DNA damage-induced 14-3-3 signaling	2.41 10^{-03}	10.5% (2/19)
**Top Upstream Regulators**	***p* values**	**Predicted activation**
IFNG	2.62 10^{-09}	Activated
TRIM24	3.63 10^{-09}	Inhibited
STAT1	1.13 10^{-08}	Activated
Ifnar	4.62 10^{-08}	Activated
IL12 (complex)	1.59 10^{-07}	No prediction

Supplementary Table 5. Top five canonical pathways and upstream regulators identified by Ingenuity pathway analysis (IPA) for the differentially expressed genes (DEGs) upregulated exclusively in adipocytes after influenza infection, relative to mock-treated controls (see Fig. 7a). The enriched pathways are ranked by *p* value and percentage of the overlapping genes mapped against the total number of those in that pathway.

GADD45 = growth arrest and DNA damage 45, IFNG = interferon gamma, Ifnar = interferon-alpha/beta receptor alpha chain, IL = interleukin.

Upregulated DEGs in preadipocytes only		
**Top Canonical Pathways**	***p* values**	**Overlap**
Superpathway of serine and glycine biosynthesis I	2.88 10^{-06}	57.1% (4/7)
tRNA charging	3.99 10^{-06}	17.9% (7/39)
Folate transformations I	1.01 10^{-05}	44.4% (4/9)
Folate polyglutamylation	4.90 10^{-05}	60% (3/5)
Glycine biosynthesis I	2.94 10^{-04}	100% (2/2)
**Top Upstream Regulators**	***p* values**	**Predicted activation**
ATF4	1.97 10^{-19}	Activated
UCP1	5.72 10^{-17}	Activated
MYC	5.33 10^{-16}	Activated
Mek	5.38 10^{-16}	Activated
PDGF BB	8.01 10^{-16}	Activated

Supplementary Table 6. Top five canonical pathways and upstream regulators identified by Ingenuity pathway analysis (IPA) for the differentially expressed genes (DEGs) upregulated only in preadipocytes after influenza infection, relative to mock-treated controls (see Fig. 7a). ATF4 = activating transcription factor 4, PDGF BB = platelet-derived growth factor binding to PDGF receptor-beta. Ranked by *p* value and percentage of the overlapping genes mapped against the total number of those in that pathway.

**Top Canonical Pathways**	***p* values**	**Overlap**
-alanine degradation I	$1.87\ 10^{-04}$	100% (2/2)
Axonal guidance signaling	$1.42\ 10^{-03}$	3.3% (15/449)
Superpathway of cholesterol biosynthesis	$6.47\ 10^{-03}$	10.7% (3/28)
Ketogenesis	$7.82\ 10^{-03}$	20.0% (2/10)
Mevalonate pathway I	$1.32\ 10^{-02}$	15.4% (2/13)
**Top Upstream Regulators**	***p* values**	**Predicted activation**
STAG1	$1.43\ 10^{-04}$	No prediction
AKT3	$1.08\ 10^{-03}$	No prediction
PRKACA	$1.08\ 10^{-03}$	No prediction
AFF1	$1.89\ 10^{-03}$	No prediction
TP53	$2.50\ 10^{-03}$	No prediction

Supplementary Table 7. Top five canonical pathways and upstream regulators identified by Ingenuity pathway analysis (IPA) for the differentially expressed genes (DEGs) downregulated exclusively in preadipocytes after influenza infection, relative to mock-treated controls (see Fig. 7a). The enriched pathways are ranked by *p* value and percentage of the overlapping genes mapped against the total number of those in that pathway.

STAG1 = stromal antigen 1, PRKACA = protein kinase cAMP-activated catalytic subunit alpha, AFF1 = AF4/FMR2 family member 1, TP53 = tumor protein 53.
