# Supplementary material for: Influenza infection rewires energy metabolism and induces browning features in adipose cells and tissues
Source: Commun Biol. 2020 May 14;3:237. doi: 10.1038/s42003-020-0965-6 (PMC7224208; doi:10.1038/s42003-020-0965-6)
Supplement: Supplementary file 8 — Description of Additional Supplementary Files [file 42003_2020_965_MOESM8_ESM.pdf]

Description of Additional Supplementary Files

File Name: **Supplementary Data 1**
Description: Ingenuity Pathway Analysis of transcriptomics of the SCAT and EWAT from IAV-infected mice vs. mock-treated mice

File Name: **Supplementary Data 2**
Description: Transcriptomics of in-vitro-IAV-infected preadipocytes and in vitro-IAV-infected adipocytes vs., respectively, in-vitro-mock-treated preadipocytes and in-vitro-mock-treated adipocytes

File Name: **Supplementary Data 3**
Description: Crude data of the morphometry analysis of the SCAT from mock-treated mice

File Name: **Supplementary Data 4**
Description: Crude data of the morphometry analysis of the SCAT from IAV-infected mice

File Name: **Supplementary Data 5**
Description: Crude data of the morphometry analysis of the EWAT from mock-treated mice

File Name: **Supplementary Data 6**
Description: Crude data of the morphometry analysis of the EWAT from IAV-infected mice
