# Supplementary material for: Influenza infection rewires energy metabolism and induces browning features in adipose cells and tissues
Source: Commun Biol. 2020 May 14;3:237. doi: 10.1038/s42003-020-0965-6 (PMC7224208; doi:10.1038/s42003-020-0965-6)
Supplement: Supplementary file 9 — Reporting Summary [file 42003_2020_965_MOESM9_ESM.pdf]

nature research

Corresponding author(s): WOLOWCZUK Isabelle

Last updated by author(s): Apr 9, 2020

Reporting Summary

Nature Research wishes to improve the reproducibility of the work that we publish. This form provides structure for consistency and transparency in reporting. For further information on Nature Research policies, see Authors & Referees and the Editorial Policy Checklist.

Statistics

For all statistical analyses, confirm that the following items are present in the figure legend, table legend, main text, or Methods section.

n/a	Confirmed	
☐	☒	The exact sample size (n) for each experimental group/condition, given as a discrete number and unit of measurement
☐	☒	A statement on whether measurements were taken from distinct samples or whether the same sample was measured repeatedly
☐	☒	The statistical test(s) used AND whether they are one- or two-sided<br>*Only common tests should be described solely by name; describe more complex techniques in the Methods section.*
☒	☐	A description of all covariates tested
☒	☐	A description of any assumptions or corrections, such as tests of normality and adjustment for multiple comparisons
☐	☒	A full description of the statistical parameters including central tendency (e.g. means) or other basic estimates (e.g. regression coefficient) AND variation (e.g. standard deviation) or associated estimates of uncertainty (e.g. confidence intervals)
☒	☐	For null hypothesis testing, the test statistic (e.g. F, t, r) with confidence intervals, effect sizes, degrees of freedom and P value noted<br>*Give P values as exact values whenever suitable.*
☒	☐	For Bayesian analysis, information on the choice of priors and Markov chain Monte Carlo settings
☒	☐	For hierarchical and complex designs, identification of the appropriate level for tests and full reporting of outcomes
☒	☐	Estimates of effect sizes (e.g. Cohen's d, Pearson's r), indicating how they were calculated

Our web collection on statistics for biologists contains articles on many of the points above.

Software and code

Policy information about availability of computer code

Data collection	BD InfluxTM cell sorter (BD Biosciences) for FACS-sorting of immune cells and preadipocytes from adipose tissues; Datlab 4 software of Oxygraph-2k (OROBOROS Instruments) for O2 consumption rates acquisition on mock-treated vs. IAV-infected preadipocytes and adipocytes (respirometry).
Data analysis	Fiji-ImageJ software (NIH) for histomorphometric analyses of the adipose tissue depots; DatLab 4 software (OROBOROS Instruments) for respirometry data analysis; Gene Ontology categories (www.geneontology.org) and Ingenuity Pathway Analyses (IPA, www.qiagen.com/) for transcriptome analyses on cells and tissues; GraphPad Prism 6 software for statistical analyses.

For manuscripts utilizing custom algorithms or software that are central to the research but not yet described in published literature, software must be made available to editors/reviewers. We strongly encourage code deposition in a community repository (e.g. GitHub). See the Nature Research guidelines for submitting code & software for further information.

Data

Policy information about availability of data

All manuscripts must include a data availability statement. This statement should provide the following information, where applicable:

- Accession codes, unique identifiers, or web links for publicly available datasets
- A list of figures that have associated raw data
- A description of any restrictions on data availability

Minimum information about Microarray Experiment (MIAME) on in vitro mock-treated vs. IAV-infected preadipocytes and in vitro mock-treated vs. IAV-infected adipocytes, was deposited in ArrayExpress database (https://www.ebi.ac.uk/arrayexpress/) under the platform ID E-MTAB-6646.
Minimum information about Microarray Experiment (MIAME) on the subcutaneous (inguinal) adipose tissue (SCAT) of mock-treated vs. IAV-infected mice, and the visceral (epididymal) adipose tissue (EWAT) of mock-treated vs. IAV-infected mice, was deposited in ArrayExpress database (https://www.ebi.ac.uk/arrayexpress/) under the platform ID E-MTAB-6646.
All data are available from the corresponding author upon request.

Field-specific reporting

Please select the one below that is the best fit for your research. If you are not sure, read the appropriate sections before making your selection.

☒ Life sciences ☐ Behavioural & social sciences ☐ Ecological, evolutionary & environmental sciences

For a reference copy of the document with all sections, see nature.com/documents/nr-reporting-summary-flat.pdf

Life sciences study design

All studies must disclose on these points even when the disclosure is negative.

Sample size	No sample-size calculation was performed. For in vivo experiments, 6 to 10 animals per group were used. Each experiment has been repeated at least 3 times. For transcriptomics (on cells and tissues) 4 biological replicates per condition were analyzed. For in vitro experiments, 3 to 4 biological replicates were done and experiments were repeated at least three times.
Data exclusions	No data were excluded from the analyses.
Replication	All in vivo and in vitro experiments were repeated at least three times, excepted transcriptomics (4 biological replicates) and TEM experiments.
Randomization	Mice were randomly assigned to be either mock-treated or IAV-infected. In the experiments of switch to high-fat diet, IAV-infected mice were randomly assigned to either remain under standard diet or to be switched to high-fat diet (Figure 4c, 4d, 4e).
Blinding	Samples were given numbers before being sent to our collaborators (supernatant testing, TCDI50 assay, cell-sorting...). In in vivo experiments, all read-outs were analyzed on individual mice with individual numbers, thus histology, morphometry, ELISA and PCR assays have been done blinded.

Reporting for specific materials, systems and methods

We require information from authors about some types of materials, experimental systems and methods used in many studies. Here, indicate whether each material, system or method listed is relevant to your study. If you are not sure if a list item applies to your research, read the appropriate section before selecting a response.

Materials & experimental systems

n/a	Involved in the study
☐	☒ Antibodies
☐	☒ Eukaryotic cell lines
☐	☐ Palaeontology
☐	☒ Animals and other organisms
☐	☐ Human research participants
☐	☐ Clinical data

Methods

n/a	Involved in the study
☐	☐ ChIP-seq
☐	☒ Flow cytometry
☐	☐ MRI-based neuroimaging

Antibodies

Antibodies used	Anti-H3N2 HA ThermoFisher Scientific Cat# PA5-34930; anti-UCP1 Abcam Cat# 10983; goat-anti-mouse IgG Alexa Fluor® 633 Invitrogen Cat# A-21052; goat-anti-rabbit IgG Alexa Fluor® 488 Invitrogen Cat# 35552; goat-anti-rabbit IgG HRP KPL Cat# 074-1506; anti-mouse CD31BV421, BioLegend clone 390; anti-mouse CD45BV510, BioLegend clone 30-F11; anti-mouse CD34PE, BioLegend clone HM34; anti-mouse CD29AF700 BioLegend, clone HMbeta1-1; Sca-1APC-Cy7, eBioscience, clone D7; anti-mouse CD16/CD32, BD Pharmingen, clone 2.4G2.
Validation	The choice of the antibodies we used has been done according to our's, and/or our collaborator's expertise.

Eukaryotic cell lines

Policy information about cell lines

Cell line source(s)	Mouse 3T3-L1 cells were bought from ATCC (CL-173TM); Human preadipocytes were bought from LONZA (Cat# PT-5020).
Authentication	Mouse and human preadipocytes were bought from companies (ATCC, LONZA).
Mycoplasma contamination	Cells were bought from companies (ATCC, LONZA). No additional tests for mycoplasma contamination were done.

Commonly misidentified lines
(See ICLAC register)

n/a

Palaeontology

Specimen provenance	n/a
Specimen deposition	n/a
Dating methods	n/a

☐ Tick this box to confirm that the raw and calibrated dates are available in the paper or in Supplementary Information.

Animals and other organisms

Policy information about studies involving animals; ARRIVE guidelines recommended for reporting animal research

Laboratory animals	Male C57BL/6JRj mice (Janvier Labs) were used (6-8 weeks of age at the beginning of experiments).
Wild animals	The study did not involve wild animals.
Field-collected samples	The study did not involve samples collected from the field.
Ethics oversight	Animals were manipulated in an Animal Biosafety Level-2 facility, in strict accordance with Institut Pasteur's guidelines on animal care and use, and in compliance with European animal welfare regulations (European Communities Council Directive of 1986 revised in 2010, 2010/63/EU). Protocols were approved by the regional Animal Experimentation Ethics Committee (Comité d'Ethique en Expérimentation Animale, Hauts-de-France, CEEA 75) and the French Ministry of Higher Education and Research (Ministère de l'Enseignement Supérieur et de la Recherche) (authorization numbers: 00357.03 and 00033.02).

Note that full information on the approval of the study protocol must also be provided in the manuscript.

Human research participants

Policy information about studies involving human research participants

Population characteristics	n/a
Recruitment	n/a
Ethics oversight	n/a

Note that full information on the approval of the study protocol must also be provided in the manuscript.

Clinical data

Policy information about clinical studies
All manuscripts should comply with the ICMJE guidelines for publication of clinical research and a completed CONSORT checklist must be included with all submissions.

Clinical trial registration	n/a
Study protocol	n/a
Data collection	n/a
Outcomes	n/a

ChIP-seq

Data deposition

☐ Confirm that both raw and final processed data have been deposited in a public database such as GEO.

☐ Confirm that you have deposited or provided access to graph files (e.g. BED files) for the called peaks.

Data access links<br>*May remain private before publication.*	n/a
Files in database submission	n/a

Genome browser session
(e.g. UCSC)

n/a

Methodology

Replicates	n/a
Sequencing depth	n/a
Antibodies	n/a
Peak calling parameters	n/a
Data quality	n/a
Software	n/a

Flow Cytometry

Plots

Confirm that:

- [x] The axis labels state the marker and fluorochrome used (e.g. CD4-FITC).
- [ ] The axis scales are clearly visible. Include numbers along axes only for bottom left plot of group (a 'group' is an analysis of identical markers).
- [ ] All plots are contour plots with outliers or pseudocolor plots.
- [ ] A numerical value for number of cells or percentage (with statistics) is provided.

Methodology

Sample preparation	Individual SCAT and EWAT were carefully excised from mock-treated and IAV-infected mice at 7 dpi. Tissues were thoroughly minced with scissors (1-2 mm3 pieces) and digested in collagenase I (1 mg/ml, Sigma-Aldrich) for 1 hour at 37°C with gentle shaking by inversion every 20 minutes. Digested tissue were filtered (250 um nylon filter) and centrifuged at 150 g for 17 minutes at room temperature. Infranatants were centrifuged at 400 g for 5 minutes at room temperature. The erythrocytes from the SVF cell-pellets were lysed with cold lysis buffer (3 minutes, on ice) and filtered through a 90 um and then a 40 um nylon filter before being washed twice by centrifugation at 400 g for 5 minutes at room temperature. Cells were counted and resuspended in PBS 0.5% BSA. SVF cell suspensions were first incubated with an Fc blocking reagent (anti-mouse CD16/CD32). cells were stained with anti-mouse CD31, CD45, CD34, CD29 and Sca1 at 1:100 dilution. Samples were then directly run into the BD InfluxTM cell sorter equipped with a 86 um nozzle and tuned at a pressure of 24.6 psi and a frequency of 48.25 kHz. Sample fluid pressure was adjusted to reach a throughput rate of 10,000 events per second. Immune cells and preadipocytes were selected as CD45+ and CD45-CD31-CD34+CD29+Sca-1+, respectively.
Instrument	BD InfluxTM cell sorter (BD Biosciences) equipped with a 86 um nozzle and tuned at a pressure of 24.6 psi and a frequency of 48.25 kHz.
Software	BD FACS™ Sortware sorter software.
Cell population abundance	Our aim was to sort adipose tissue immune cells (selected as CD45+ cells) and preadipocytes (selected as CD45-CD31-CD34 +CD29+Sca-1+ cells) from the adipose tissues. Sorted cells were immediately stained with anti-HA antibody.
Gating strategy	The gating strategy that was used is provided in Supplementary Figure 5b. Stromal vascular faction (SVF) cell suspensions were prepared from the SCAT and EWAT of mock-treated and IAV-infected mice (individually), 7 dpi, n = 3 mock and n = 6 IAV. Immune cells were identified as CD45+ cells. Preadipocytes were identified as CD45- CD31- CD29+ CD34+ Sca1+ cells.

- [x] Tick this box to confirm that a figure exemplifying the gating strategy is provided in the Supplementary Information.

Magnetic resonance imaging

Experimental design

Design type	n/a
Design specifications	n/a
Behavioral performance measures	n/a

Acquisition

Imaging type(s) | n/a

Field strength | n/a

Sequence & imaging parameters | n/a

Area of acquisition | n/a

Diffusion MRI ☐ Used ☐ Not used

Preprocessing

Preprocessing software | n/a

Normalization | n/a

Normalization template | n/a

Noise and artifact removal | n/a

Volume censoring | n/a

Statistical modeling & inference

Model type and settings | n/a

Effect(s) tested | n/a

Specify type of analysis: ☐ Whole brain ☐ ROI-based ☐ Both

Statistic type for inference (See Eklund et al. 2016) | n/a

Correction | n/a

Models & analysis

n/a	Involved in the study
☒	☐ Functional and/or effective connectivity
☒	☐ Graph analysis
☒	☐ Multivariate modeling or predictive analysis
